# Congenital aortic arch abnormality in tetralogy of Fallot: A rare case report: Retraction

**DOI:** 10.1097/MD.0000000000044338

**Published:** 2025-08-22

**Authors:** Rakshand Shetty, Hashim Talib Hashim, C. S. Madhu, Akash Uppin, Rupali Sachdev, V. Ashish Chandra, Jyothika Venkatswamy Reddy, Ashraf Fhed Mohammed Basalilah, Lamees Abd AlRaheem Nabat

The article “Congenital aortic arch abnormality in tetralogy of Fallot: A rare case report”^1^, which appears in Volume 104, Issue 11 of *Medicine*, is being retracted. Information brought to our attention during a recent investigation casts doubt on both the authorship and credibility of the presented case, and the Publisher has lost faith in the integrity of the content.
